# Genetic characterisation of influenza C viruses detected in Singapore in 2006

**DOI:** 10.1111/irv.12352

**Published:** 2015-12-11

**Authors:** Pei‐jun Ting, Shirley Gek‐Kheng Seah, Elizabeth Ai‐Sim Lim, Jasper Chin‐Wen Liaw, Boon‐Huan Tan

**Affiliations:** ^1^ DMERIDSO National LaboratoriesSingaporeSingapore

**Keywords:** Febrile respiratory illness, influenza C virus, phylogenetic analysis, Singapore military recruits

## Abstract

In an earlier study on respiratory infections in Singapore military recruits, four influenza C virus (FLUCV) infections were detected out of the 1354 samples collected. All four isolates were detected in 2006, and their whole genome was completely sequenced and analysed. Phylogenetic analysis of the hemagglutinin esterase fusion (HEF) gene revealed that all four Singapore isolates belonged to the C/Japan‐Kanagawa/1/76‐related lineage. However, the genes of the four FLUCV isolates had origins from several different lineages, and the genome composition resembles that of the C/Japan‐Miyagi/9/96‐like strains that had been circulating in Japan between 1996 and 2000.

## Introduction

Military personnel generally have increased vulnerability to respiratory infections, and these have been attributed to different challenges in military lifestyle.^1^ Outbreaks of respiratory diseases have previously been reported in military populations. For example, the predominant cause of febrile respiratory illness (FRI) in US military recruits has consistently been human adenoviruses.^1^ Other virus infections such as influenza A virus (FLUAV) outbreaks and influenza B virus (FLUBV) infections have also been documented in the Taiwan military personnel.^2^ In the Singapore military recruits, the main causative agent for FRI was reported to be FLUAV/FLUBV, at an incidence of 36% (485/1354).^3^ Although influenza A, B and C viruses belong to the same *Orthomyxoviridae* family, current epidemiological research tend to focus more on FLUAV and FLUBV. Outbreaks of influenza C virus (FLUCV) infections have been detected mainly in Japan and reported elsewhere for example in France.^4^, ^5^, ^6^

Little is known about the epidemiology of FLUCV circulating in a tropical climate in Singapore, in either the military or civilian population. The same FRI surveillance study conducted on the Singapore military recruits in 2006–2007 was also able to detect four cases of FLUCV infection.^3^ FLUCV usually causes mild upper respiratory tract illness and its genome comprises of seven RNA segments encoding for nine different proteins – PB2, PB1 and P3 polymerase proteins, hemagglutinin esterase fusion (HEF) protein, nucleoprotein (NP), CM1 and CM2 matrix proteins and NS1 and NS2 non‐structural proteins.^4^ In this first study on FLUCV in Singapore, we performed genetic characterisation of the four Singapore FLUCV isolates by sequencing all seven RNA segments from the four isolates and examining their genetic relatedness with existing FLUCV sequences.

## Materials and methods

### Virus Isolation and nucleic acid extraction

The viruses were first isolated in Madin–Darby Canine Kidney cells with throat swab specimen collected from military recruits as described previously.^3^ Cultures were maintained in Dulbecco's modified Eagle's medium (ThermoFisher Scientific Inc, Waltham, MA, USA) supplemented with 2 μg/ml of TPCK‐trypsin and 0·1% of bovine serum albumin at 33°C till cytopathic effect was observed. Viral RNA extraction was then performed on the harvested culture supernatant using the QIAamp viral RNA mini kit (Qiagen, Inc., Valencia, CA, USA) according to the manufacturer's instructions.

### Nucleotide sequencing

Reverse transcription of the vRNA was performed using the Transcriptor First Strand cDNA Synthesis Kit (Roche Diagnostics, Mannheim, Germany) with the Uni12 primer.^7^ The seven RNA segments from the FLUCV were PCR amplified using primers described by Crecenzo‐Chaigne *et al*.,^8^ and Kimura *et al*.^9^ Full‐length genes from the FLUCV isolates were obtained by performing PCR using overlapping primer pairs and sequencing with the ABI Prism BigDye (ThermoFisher Scientific Inc). The sequences were assembled using the SeqMan software (DNASTAR, Lasergene version 7, Madison, WI, USA). All sequences were submitted to GenBank and assigned the accession numbers GQ853452‐55 and JX963106‐29.

### Phylogenetic analysis

Nucleic acid sequences obtained from the Singapore isolates were aligned with existing sequences published in the GenBank database using the MegAlign software (DNASTAR, Lasergene version 7, Madison, WI, USA). Phylogenetic trees were constructed by the neighbour‐joining method using bootstrap values of 1000 replicates.

## Results

**Figure 1:**
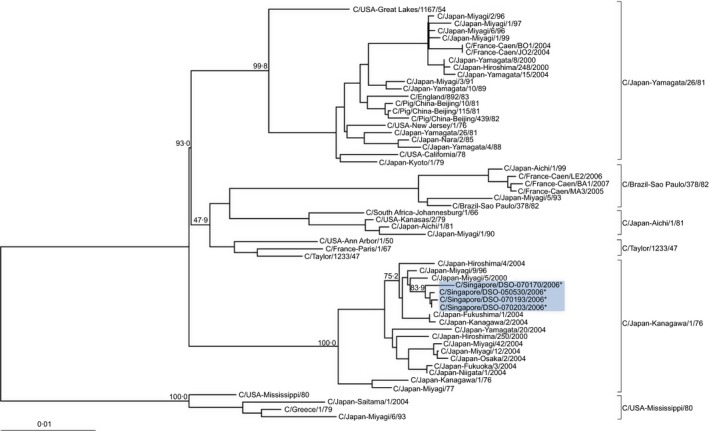
Phylogenetic tree of the HEF genes from human and swine FLUCV isolates based on nucleotide sequence alignment. The tree was generated using the neighbour‐joining method, bootstrapped with 1000 replicates and viewed with TreeView. Numbers above the branches are the bootstrap values determined by the MegAlign software. The HEF genes of the Singapore strains sequenced in this study are highlighted by asterisks and shaded in blue. The sequences of the other FLUCV viruses were obtained from the Genbank database. Sequences belonging to swine isolates have host information as part of the sequence names. Sequence names without host information are human FLUCV isolates.

All seven RNA segments (PB1, PB2, P3, NP, HEF, NS and M) from the four Singapore FLUCV isolates were sequenced, and phylogenetic analysis was performed with published sequences in GenBank. Previous reports have categorised the HEF gene from FLUCV into six distinct lineages represented by the C/Japan‐Kanagawa/1/76, C/USA‐Mississippi/80, C/Taylor/1233/47, C/Japan‐Aichi/1/81, C/Japan‐Yamagata/26/81 and C/Brazil‐Sao Paulo/378/82 strains.^4^, ^5^, ^10^ The nucleotide and amino acid sequences of the HEF genes from these four Singapore FLUCV isolates were highly similar, with sequence identities of 99·5–99·9% and 99·3–100%, respectively. Phylogenetic analysis performed on the HEF gene nucleotide sequences revealed that the four Singapore FLUCV isolates clustered together in the same branch under the C/Japan‐Kanagawa/1/76‐related lineage (Figure 1).

**Figure 2:**
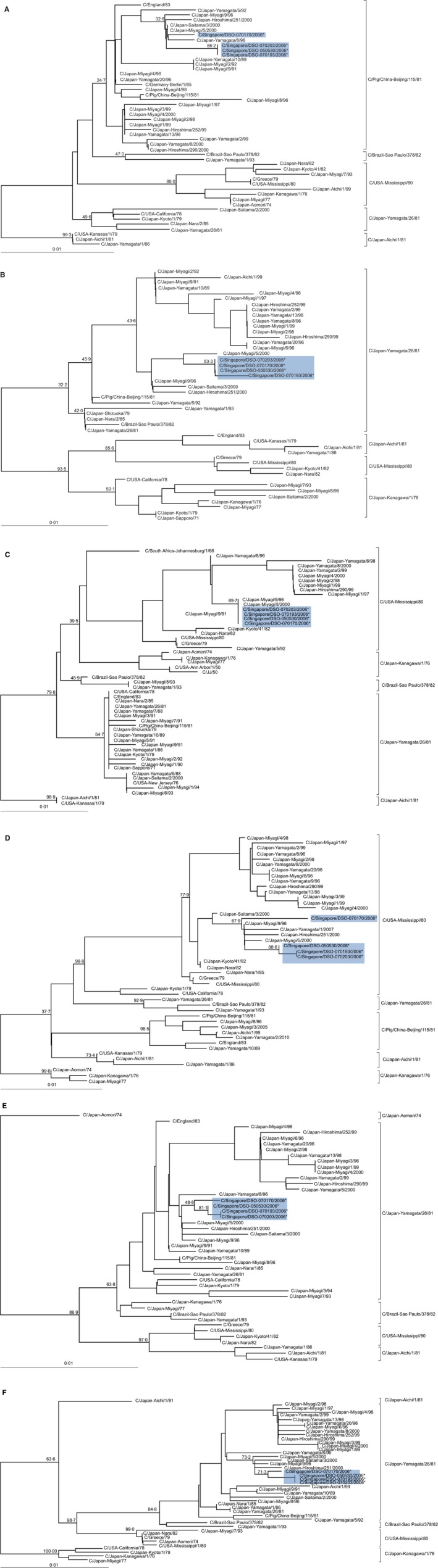
Phylogenetic trees of the PB2 (A), PB1 (B), P3 (C), NP (D), M (E), NS (F) genes from human and swine FLUCV isolates based on nucleotide sequence alignment. The trees were generated using the neighbour‐joining method, bootstrapped with 1000 replicates and viewed with TreeView. Numbers above the branches are the bootstrap values determined by the MegAlign software. The genes from the Singapore strains sequenced in this study are highlighted by asterisks and shaded in blue. The sequences of the other FLUCV viruses were obtained from the Genbank database. Sequences belonging to swine isolates have host information as part of the sequence names. Sequence names without host information are human FLUCV isolates.

Phylogenetic trees for the other six RNA segments were also generated and we also observed the same clusters described in earlier publications.^5^, ^11^, ^12^ While all four Singapore isolates clustered closely together on the individual trees, it was observed that the genes of these four isolates had origins from several different lineages (Figure 2). The PB1, M and NS genes clustered into the C/Japan‐Yamagata/26/81‐related lineage, PB2 gene into the C/Pig/China‐Beijing/115/81‐related lineage, and P3 and NP genes into the C/USA‐Mississippi/80‐related lineage.

## Discussion

Influenza C virus is often neglected due to its low disease severity. However, the fact that it continues to circulate at background levels as presented by Seah *et al*.^3^ in conjunction with FLUAV and FLUBV indicates its ability in causing disease. The four FLUCV isolates studied in this report were isolated from recruits undergoing military training. Clinical symptoms presented by these four patients include fever of ≥38°C, cough, nasal congestion and body ache which lasted for 1–2 days. While not extremely severe, these recruits suffered from illnesses serious enough to seek medical attention which resulted in the loss of man hours, something which cannot be afforded in a military setting. It was also recently reported by Kauppila *et al*.^13^ that FLUCV is an important respiratory tract infection amongst army conscripts in Finland.

The four FLUCV isolates characterised in this report were collected in a FRI surveillance study carried out from March 2006 to April 2007. The FLUCV samples were detected in the months of May, November and December. Since the Singapore FLUCV isolates were collected within months of each other, it is likely that they would be highly related to each other. As expected, the PB1, P3, HEF, M and NS genes from all four isolates showed high nucleotide sequence identities ranging from 98·8% to 100%. The only exceptions were the PB2 and NP genes of the C/Singapore/DSO‐070170/2006 strain, which clustered away from the other three Singapore FLUCV strains for these genes in the phylogenetic trees. It may suggest that this particular strain may be a variant of the other Singapore FLUCV strains, especially in terms of its NP gene, which differs from the NP genes of the other three Singapore FLUCV strains by up to 1·2%.

All four Singapore FLUCV strains isolated in 2006 were classified into the C/Japan‐Kanagawa/1/76‐related lineage based on the HEF gene. In this same time period, the dominant FLUCV strain circulating in Japan belonged to the C/Brazil‐Sao Paulo/378/82‐related lineage,^6^ while in Caen, France, two dominant strains of the C/Brazil‐Sao Paulo/378/82 and C/Japan‐Yamagata/26/81‐related lineages were found to be cocirculating.^4^ This suggests that the worldwide distribution of FLUCV may vary, with each geographical location presenting a different predominant strain in the same timeframe. It was also not uncommon for FLUCV belonging to multiple distinct lineages to cocirculate at the same time.^10^ Although not observed in the Singapore context, the present surveillance study was only conducted on the military population and does not give a representation of the Singapore population as a whole.

Phylogenetic analysis of the genomes of the Singapore FLUCV isolates found that these isolates were made up of genes originating from several different lineages. This suggested that the Singapore isolates may have went through several rounds of reassortment. Earlier publications had reported the high frequency of reassortment events between different strains of FLUCV both *in vitro* and in nature.^10^, ^14^ The genome composition of the Singapore FLUCV isolates was identical to the C/Japan‐Miyagi/9/96 strain described by Matsuzaki *et al*.,^10^ with the HEF gene clustered to the C/Japan‐Kanagawa/1/76‐related lineage. The P3 and NP genes clustered to the C/USA‐Mississippi/80‐related lineage, the PB2 gene to the C/Pig/China‐Beijing/115/81‐related lineage, and PB1, M and NS genes to the C/Japan‐Yamagata/26/81‐related lineage. Moreover, the nucleotide sequence identities of the four Singapore FLUCV strains and the C/Japan‐Miyagi/9/96 strain ranged between 99·1% and 100% for all seven genes, putting them together as highly similar strains. This genetic relatedness to the C/Japan‐Miyagi/9/96 strain suggested an association of the Singapore FLUCV strains to the Japanese strain and that the FLUCV circulating in Singapore may have been imported. The C/Japan‐Miyagi/9/96 strain was reported to have gained advantage in the natural selection process via a reassortment event with the C/Japan‐Yamagata/26/81‐related strains which had caused epidemics in Japan in 1996 and 1998.^10^ This enabled the first C/Japan‐Kanagawa/1/76‐like strain to re‐emerge in Japan after an absence of 20 years, from 1977 to 1995.^10^ This selection fitness may have also allowed the virus to continue its spread overseas, enabling its highly similar strain to be detected in Singapore ten years after its initial re‐emergence.

This report presents the first study on FLUCV in Singapore. Carried out in the military population, it provides a limited scope of the epidemiology of FLUCV circulating in a tropical climate in Singapore. Continuous surveillance of FLUCV in both the military and civilian population in Singapore will therefore be critical to provide a clearer epidemiological trend and clinical significance of the FLUCV circulating in the country.
